# Medical Items and News of Interest to the Physician

**Published:** 1883-10

**Authors:** 


### Medical Items and News,

### For the Use of Physicians.

CULLED FROM THE STANDARD MEDICAL JOURNALS OF THE WORLD.

NOTE.—These formulæ are intended only for the regular practitioner of medicine, and should be employed only by him, or under his immediate supervision.

**Antiblenorrhagic Boli.**

The following is a formula for the much praised "antiblenorrhagic bolus" of Simmonot, of Paris:

℞

Copaiba balsam, 25 grm.
Powd. cubebs, 122 grm.
Wax, 12 grm.
Rhatannia powder, 9 grm.
Carb. magnesia, 6 grm.

The boli are made to weigh 1 grm. each, and when finished they are rolled in subcarb. iron, and coated with an ethereal solution of balsam tolu and mastich.—*Journal de Medecine de Paris.*—*Ther. Gaz.*

**Guachamaca.**

Dr. Carl Sachs brought this plant from Venezuela to Europe, and prepared an extract from it. Its action is similar to that of curare; besides it has the power of producing sleep, but this effect makes its appearance very late; in one instance, in Prof. Frerich's clinic, after the injection subcutaneously of 11 milligrams the patient fell asleep three-quarters of an hour after the administration of the extract. The hypnotic effect continued for fully three hours, without any change in the circulation or respiration.—*Monatsschrift f. Deut.-Amer. Aerzte.*—*Ther. Gaz.*

**Hyperidrosis and Urticaria.**

Atropine is a sovereign remedy to arrest the profuse sweating of phthisical patients. Schwimmer obtained good results also from its use in local and universal hyperidrosis. In some of the most obstinate forms of urticaria the use of atropine is very satisfactory.

℞

Atropiæ sul. 0.008.

Glycerinæ.

Aquæ ana 2.0.

Gum Trag. q. s.

M. Ft. Pills No. xvi.

The dose may range from ½ to ¾, mgrm, pro die given until symptoms of intoxication make their appearance.—*Zeitsch. f. Therapie.*

**Burns.**

At various times during the past eleven years, there has been a method for treating *burns* in use in the public hospitals of the city of New York which was originally devised by one of the physicians, and which closely resembles the glycerin bandage. It is known as "Glue Burn Mixture:"

White glue, troy oz. 7½.
Water, fl. oz. 16.
Glycerin, fl. oz. 1.
Carbolic acid, fl. dr. 2.

Soak the glue in the water until it is soft; then heat on a water-bath until melted; add the glycerin and carbolic acid, and continue heating until, in the intervals of stirring, a glossy, strong skin begins to form over the surface. When wanted for use, heat on a water-bath, and apply with a flat brush over the burned part.

**Convallaria Majalis.**

In cases of organic cardiac troubles, especially in insufficiency of the mitral valve, extract convallariæ majalis aq. proved itself to possess marked stimulating and diuretic properties. As a tonic and diuretic in feeble heart as in kidney troubles its action is not so evident. In compensatory derangements of the valves it is unexcelled. The author found no evil results even after larger doses.—*Cbl. f. Therapie.*—*Ther. Gaz.*

**Ulcers of the Tongue.**

J. Paget advised the use of a concentrated solution of chromic acid in psoriasis of the tongue. Butlin used a solution (0.5 to 100) by means of a brush in the different varieties of ulcer of the same organ. The effect of chromic acid in secondary syphilitic ulceration was highly satisfactory, while the rhagades of the tertiary stage healed very kindly.—*Wiener Med. Blat.*

**Colic.**

Dr. N. R. Derby, of Bergen Point, N. J., writes in the *Medical Record* that he discovered, by accident, that a dose of eight to ten grains of sulphate of quinine will speedily put an end to an attack of colic. Since childhood he had himself been a frequent sufferer from this distressing trouble, but since this discovery he has been able to arrest an attack either in himself or others. His observations extend over a period of three years, and have embraced many cases.

**New Remedy for Asthma.**

Euphorbia pilulifera is administered in Australia for asthma, and is said to be superior to all others. It is prepared as follows: Thirty grains of the leaves are boiled in two liters and 270 grams of water, which is reduced to 560 grams. Of this, from two to three glassfuls are drunk daily.—*Med. Rec.—Jour. de Medicine, Centralblatt fur Therapie.—Ther. Gaz.*

**Excessive Sweating.**

One drachm of sulphate of quinine is to be dissolved in a pint of alcohol, and the body and limbs are to be bathed with it, a small surface at a time being exposed to prevent catching cold by evaporation. Dr. T. H. Currie, of Lebanon, N. H., is said to have found this efficacious during an experience of tnirty years.

**Acute Infantile Rheumatism.**

℞

Quiniæ sulphatis, grs. xlvj.
Veratriæ. gr. $\frac{1}{8}$.
Extracti digitalis, gr. iij.

Misce et ft. pilulæ, No. xxv.
Sig.—From eight to ten daily.

*Petit Moniteur de la Medicine.—Ther. Gaz.*

**Rheumatic Fever.**

H. Greenway, R. C. S., writes: "The sulpho-carbolate of soda has acted admirably in cases of rheumatic fever. For adults I prescribe 15 grains every six hours in one ounce and a half of water. Ordinary precautions of administering an occasional aperient, placing the patient between blankets and keeping him on milk diet, must not be neglected."

**Hydrastis Canadensis.**

The root of this ranunculacea contains berberin and hydrastin. The fluid extract prepared from the root without alcohol is highly recommended in all pathological conditions of the mucous membranes, especially as an injection in gonorrhœa and leucorrhœa, etc. When it is to be administered internally in dyspepsia, chronic affections of the stomach, the following formula is a good one:

℞

Fl. ext. canadensis, 2 parts.
Sherry wine, 12 parts.
Alcohol, 1 part.

M. D. S. One-half to one wineglassful three to four times a day.

The white alkaloid of hydrastis can., called hydrastin, seems to expend its action mainly on the mucous surfaces, while berberin brings about the tonic effect.—*Pharmaceutische Post.*

**Diuretic Tea.**

Cream of Tartar, 15 parts.
Borax, 10 parts.
Lovage root (*Liguzticum Levisticum*), 25 parts.
Restharrow herb (*Ononis Spinosa*), 25 parts.
Sassafras bark, 25 parts.

Coarsely ground and mixed. A tablespoonful boiled with three cupfuls of water down to two cupfuls.—*New Idea.*

**Catarrh.**

The following combination is stated by M. P. Vigier to have been used with good success in catarrhal affections:

℞

Picis liquidæ, ...... 1.0 gm. 15=grs.
Benzoini .......... 1.0 " 15 "
Pulv. ipecac et opii 1.0 " 15 "

Mix. Make into ten pills. Take three pills daily between meals.

**Cystitis.**

Dr. J. Andeer (*Centralbl. f. die Med. Wissen.,*) reports extensive use of resorcin in acute and chronic cystitis, and claims for it almost specific curative power. He reports 156 cases where, either by him or to his personal knowledge, it was injected into the human bladder with the best results in vesical catarrh Acute cases have been entirely cured by the injection of a five-per-cent solution.—*Am. Journ. of the Med. Sci.*

**Erysipelas.**

Phenic acid, 1 part.
Alcohol, 1 part.
Essence of turpentine, 2 parts.
Tincture of iodine, 1 part.
Glycerine, 5 parts.

With this moisten all superficial erysipelatous patches every two hours.—Rothe, *Revista Pharmaceutica.*

**Diethylacetate and Dimethylacetate as Anæsthetic.**

Dr. Mering, at a recent meeting of German naturalists and physicians, reports experiments with two anæsthetics of the ethyle and methyl series. The former has a burning, pungent taste, the latter a disagreeable taste and smell. Both produce narcosis rapidly in frogs and rabbits, with slowing of the heart's action, and, finally, weakening of respiratory movements, acting, when inhaled, much like chloroform. When the former was tried on criminals it acted well, causing narcosis without bad after-effects.

**Dyspepsia.**

Dr. G. Kerlus argues from the facts of comparative physiology that fine sand is a good thing for dyspeptics to take with their food. Herbiferous animals all eat a little dirt with heir regular food, and it makes it more "porous." Fowls and birds of all kinds also take sand with their meals. "Why not, therefore, man?" says Dr. Kerlus. Putting this brilliant piece of inductive reasoning into practice, he has administered finely ground sand with the food of his patients, and, of course, reports cures.—*Medical Record.* [Why not? Perhaps the answer will be readily found in the circumstance of man being deprived of a gizzard, in which to utilize the sand for its "grinding" properties.—ED. BISTOURY.]

---

**Dry Treatment of Otorrhœa.**

Dr. Burnett (*American Journal of Medical Sciences*) holds that the syringe should be used in this disease only when absolutely necessary for the removal of accumulation, and then only by the physician. Warmth and moisture favor the fungoid growths. The home treatment should consist of drying the ear with a twisted pencil of absorbent cotton. The physician should blow into the ear dry powders. For this, Dr. B. recommends the following: Triturate equal parts (grain to the minim) of boracic acid and tincture of calendula officinalis; allow to evaporate, and rub one part of this with from one to two parts of pure boracic acid. The author holds that recovery takes place under this treatment in one-sixth the time required by the wet treatment.

---

**Submammary Intercostal Neuralgia.**

J. Chéron states that a good many women suffer from a localized pain in the left inframammary region, which is due to uterine disease. In some cases it is so painful and persistent that the primary affection may be overlooked. More or less success has attended the use of quinine, aconite, morphine, atropine, the bromides, etc. Chéron has had good results from the use of the following: Tincture gelsemium gtt. c, simple syrup ℥ ij, distilled water ad ℥ viij. S.—Dose, three to five dessert-spoonfuls half an hour before meals, or three hours after. Sometimes there are extremely painful spots in the course of a nerve, and running back to the spine. These may be painted with tincture of iodine every three days.—*Progres Med.*

**Heart Disease.**

A single drop of the tincture of digitalis, given to a patient suffering from symptoms due to heart disease, where digtialis is indicated, administered at intervals of an hour, or half an hour, according to the severity of the symptoms, will often give greater relief than larger doses, and without liability to ill effects.

---

**Scarlet Fever.**

Grain doses of chlorate of potassium, given every half hour, in scarlet fever, diphtheria, tonsilitis, etc., will produce the same results as larger doses, without the danger of the evil effects resulting from the accumulation of the drug in the system, as sometimes happens when it is administered in the ordinary way. Indeed, they will produce better results upon the inflammations of the throat.

---

**Neuralgia.**

Croton chloral, when used to relieve neuralgia, is more efficacious when a grain is administered every half hour until the symptoms abate than when, as is usually the case, five to eight grains are repeated every two hours until fifteen grains are taken. Another advantage of the former plan is that it avoids irritation of the stomach, so apt to be caused when the larger doses are used.

---

**Nasal Catarrh.**

Cubeb is the remedy most relied on in the throat room, for constitutional impression in the ordinary form of the complaint. Fifteen or more drops of the oleo resin, on sugar, after meals; or a few grains of the recently prepared powder, with two or three grains of salicylate of cinchonidia, in pill or capsule, are the forms in which it is usually prescribed. Cleanliness, by douche or spray, is essential in giving the parts a chance to get well, which they often do by cleanliness alone, without any topical medication.—*The Polyclinic.*

---

**Impaction of Cerumen.**

Dr. George P. Sowers (*Medical and Surgical Reporter*) gives the following formula for dissolving impacted cerumen, so that it can be removed with the syringe:

℞

Sodii boratis, pulv., ℥ j.

Glycerine, }

Aquæ, } āā., ℥ ii.

M. Sig. Warm, and drop into the ear. After it has liquefied the cerumen, use syringe and tepid water.

**Facial Neuralgia.**

Three-minim doses of the tincture of gelsemium every half hour will often relieve miraculously, neuralgias about face or head, and leave no ill effects.—*Prof. Smith, Bellevue Med. Col.*

**Iodoform, Covering the Odor of.**

A correspondent of the *Pharmaceutische Zeitschrift fur Russland* states that the following combination completely covers the odor of the iodoform:

Iodoform, gm. 4 = 60 gr.
Vaseline or lard, gm 30 = 1 oz.
Naphthalin, gm. 0.3 = 5 gr.
Oil of peppermint, gtt. 10 = 10 gtt.

**Creosote Wine.**

Wood creosote, 6 parts.
Compound Tincture of gentian, 30 parts.
Alcohol, 230 parts.
Sherry wine, 710 parts.

Mix. Dose, a tablespoonful two or three times daily in a cupful of water.

Recommended in phthisis to diminish cough, fever, expectoration, etc.

**Alterative Tonic.**

Goodell's "four chlorides mixture" is composed as follows:

℞

Hydrarg. bichlor, gr. j-ij.
Liq. arsen. chlor., ʒ j.
Acidi. hydrochlor, dil.
Tr. ferri. chlor., of each, ʒ ij.
Syr. Zingib, ℥ ij.
Aquæ ad., ℥ vj.

M. Sig.—Two teaspoonfuls three times daily in water, after meals.

This is prescribed by Prof. Goodell as an alterative tonic.

**Micro-organism of Pneumonia.**

The authors present a special micro-organism (different from any yet discovered) which they found both in the sputa and blood of patients with pneumonia. The sputum, injected subcutaneously in rabbits and dogs, produced "fatal acute septicemia." Defibrinated blood of patients with pneumonia injected into the peritoneal cavity of dogs produced only temporary fever. In no case did genuine pneumonia follow the injections. In some instances the culture-fluid was inserted in the trachea itself, producing, however, only acute septicemia.—L. Griffini e A. Cambria, *Esta. dal. Gior. internaz delle sc. med. iv.*

**Diabetes Mellitus.**

Massoin claims to have observed frequent good results from the use of permanganate of potassium in diabetes mellitus, and he ascribes the entire action to the manganese, because according to Laschkewitz it produces a fatty metamorphosis in the liver, and because in many cases the liver participates, especially in the case of men.

Therefore it is these hepatogenic cases, in Massoin's opinion, that are favorably influenced by the manganate.—E. Massoin, *Bull. de l'acad, royale de med. de Belgique.*

**Squint Cured Without Operation.**

In the early stages of convergent strabismus, before the internal rectus muscle is permanently contracted, Dr. Boucheron (*Schmidt's Jahrbucher*), claims that a cure is possible without operation. He states that as convergence is caused by efforts of accommodation for near objects, if we take away the power of accommodation squint will not occur. He maintains a constant mydriasis by the instillation of atropine night and morning. A cure is usually obtained in two or three weeks. If atropine is not well borne, other mydriatics, such as duboisia, may be used. In nine cases of intermittent strabismus the author obtained eight cures by this method.—*Med. Record.*

**Fetid Foot-Sweat.**

Dr. Armaingaud has used a hypodermic injection of pilocarpine in several cases of fetid foot-sweat with good results. The suppression of sweating about the feet, even when rapidly brought about by the use of this remedy, does not appear to affect the general organism injuriously. Whether the effect is permanent cannot be decided at present. Pilocarpine acts here by exciting a diverting secretion in the salivary glands; the sudorific effect, which is more readily obtained with jaborandi than with pilocarpine, does not appear to be able to replace the specially salivating effects of the latter.—*Pharm. Post.*

[Special attention may here also be directed to the difference in therapeutic effect between jaborandi and its alkaloid. Jaborandi is both a sudorific and saliva-increasing drug; the alkaloid pilocarpine, however, appears to possess the last-named property in much greater proportion, a fact which has been noticed by many careful observers. Probably some of the other principles obtained in jaborandi, perhaps *jaborine*, are more energetic in the other direction.]

**Delirium Tremens.**

Dr. Arnoldon has used ergot in several cases of mania-à-potu, with the effect of speedily controlling the delirium. He explains its action by its influence in contracting the cerebral blood-vessels.—*Med. Record* from *Deutsche Med. Zeit.*

---

**Boils.**

Dr. Gourgues (*Journal de Med. de Paris*) recommends an ointment of four parts of finely powdered boracic acid, one-half part of benzoic acid, and twenty parts of vaselin. It promptly relieves pain, and causes the disappearance of the boil in three or four days.

---

**Piles.**

Capsicum, Vidal regards as the best remedy in piles. He prescribes three or four 3-grain pills daily, half at breakfast time and half at supper time. Under its influence congestion and all the painful symptoms which accompany it, disappear rapidly.

---

**Diphtheria.**

A writer in the *Medical and Surgical Reporter* highly recommends the topical application of iodoform in diphtheria. He says that the fetor is completely obliterated and the disease very much abbreviated. It is recommended to dust it on the membrane once or twice daily.

---

**Spasmodic Cough.**

Dr. William Squire recommends a solution of one part of bromide of ethyl in two hundred parts of water as a remedy for whooping-cough, and also in angina pectoris. This is of similar strength to the chloroform water of the British Pharmacopœia, and its dose is the same, viz., one-half to two ounces.

---

**Emmenagogue Mixture.**

Dr. C. H. Wood, in his Therapeutics and Materia Medica, says: The following formula, known as Dewee's Emmenagogue Mixture, I rely upon almost exclusively in the treatment of simple atonic amenorrhæ. The amount of iron should be as the anæmia, of aloes as the state of the bowels, of cantharides as the susceptibility of the urinary organs:

℞

Tincturæ ferri chloridi, f ʒ iii.

Tincturæ cantharidis, f ʒ i.

Tincturæ aloes, f ℥ j.

Tincturæ guaiaci ammoniatæ, f ℥ iss.

Syrupi, x. s., ad., f ʒ vi.

Sig. Tablespoonful three times a day.

**A Good Vermifuge.**

Iodoform in doses of one grain is said to be a good vermifuge.

---

**Migrains.**

One grain of citrate of caffeine every half hour often produces most marked relief in migrains.

---

**Lumbago.**

Lumbago may be quickly relieved (*Scientific American*) by binding a piece of oil-skin cloth, such as is used to cover tables, over the loins outside of the flannel shirt. Profuse perspiration is produced, which rapidly relieves the pain.—[What does the *Scientific American* know about it, anyway ?—ED. BISTOURY.]

---

**Phthisis.**

Mr. H. Osborn Bayfield suggests (in the *Brit. Med. Journ.*) that the use of inhalations of volatilized palm oil may be useful in the treatment of phthisis. He bases his opinion upon the fact that workmen engaged in tinning where palm oil is used as a flux, inhale the volatilized oil and get fat. Those previously emaciated or weak rapidly improve. The idea is worth a trial.

---

**Sweats of Phthisis.**

Dr. Landouzy employs a powder consisting of 10 parts, by weight, of salicylic acid to 90 of talc or starch. Those parts of the body which are habitually the most frequent seats of the sweating are powdered twice a day. Almost always it gives temporary relief, and sometimes the amelioration persists for some days after the application has been discontinued.—*Journal de Therapeutique.*

---

**Soft Chancres and Buboes.**

The efficacy of salicylic acid in the treatment of soft chancres and of buboes appears to us to be unquestionable. While not an absolute specific, it is, in our opinion, capable of being most advantageously employed.

Odorless, only slightly painful in its application, soluble in alcohol and in glycerine, and leaving no stain on linen, it is preferable, in these important respects, to most other agents employed for the cure of the above named affections, while perhaps inferior in certain other particulars to some among its rivals.

It may be resorted to in all cases, and is equally available in private and hospital practice.—*Autier, Th. de Paris.*

**Eczema.**

The following application is recommended in the *Zeitschr. f. Therapie:*

Boracic acid in very fine powder, 6 gm.=90 grs.
Balsam of Peru, 0.5 gm.=8 grs.
Vaseline, 30 gm.=1 oz.

**Soda Mint.**

Bicarbonate of soda, 4 drachms.
Aromatic spirit of ammonia, 1 ounce.
Spearmint or peppermint water, 16 oz.

Mix. Dose, from a dessertspoonful to a tablespoonful for adult..

**Teething Infants--Bromide of Sodium.**

A few grains dissolved in a tumblerful of water, so that each teaspoonful may represent a half grain, will quickly quiet the nervous disturbance of teething infants, or fever not dependent upon the onset of an inflammation or other grave trouble, but rather such as may follow excitement of any kind. The dose should be repeated every ten or fifteen minutes.

**Glycerite of Starch.**

The following formula for glycerine ointment is given by Vulpius in the *Pharmaceutische Zeitung*, and is recommended as giving a better preparation than those made according to the old or new German Pharmacopœia:

Tragacanth, powdered, 1 part.
Alcohol, 5 parts.
Starch, 10 parts.
Distilled water, 10 parts.
Glycerine, 100 parts.

**Hæmoptysis.**

The milk-thistle *carduss marianus* is said by Dr. Lesenwitch to be serviceable for the relief of hæmoptysis, he having tried it in five cases, using the tincture in doses of fifteen to twenty drops in a teaspoonful of water every two hours. In two of these cases where pulmonary cavities existed, it was inferior to ergot.—*Jour. de Med. de Paris.* [Other names given to this plant are *Silybum Marianum* Gaertn., *S. maculatum* Monch., *Circium maculatum* Scop., *Carthamus maculatus* Lamb., *Carduus Mariæ, Cnicus marianus*, Our Lady's thistle, and St. Mary's thistle. Flowers purple, July, perennial, waste places. It is a native of Southern Europe. Its properties are said to be pectoral, antipleuritic, aperitive. Full-grown leaves are said to be sudorific and aperient. Alphonso Wood does not mention it among the thistles indigenous to this country.]

**Neuralgia.**

Chloroform is a powerful remedy in neuralgia. It is used almost exclusively locally, occasionally, however, by inhalation in very severe cases. It gives especially good results when used hypodermically. Such injections should be made deeply into the cellular tissue or muscular interstices of the painful region. Plunge your needle then, perpendicularly, into the tissues, and carry it as far as the guard at its proximal extremity. This mode of treatment is hardly applicable to any form of neuralgia except sciatica.

**Iodoform—Solution for Hypodermic Use.**

Mosetig gives the following directions, in the *Zeitschr. f. Therapie*, for preparing a solution of iodoform for hypodermic use:

Iodoform, gm. 1.0=15 grs.
Benzol, gm. 3.0=45 grs.
Vaseline oil, gm. 15.0=240 grs.
Oil of gaultheria, gtt. 2=2 gtt.

We have no practical experience with this mixture, but confess that we would not like to adopt this form of administration without feeling our way cautiously, on account of the possibility of producing abscess.—*New Remedies.*

**Chorea.**

James Sawyer, M. D., in the course of a clinical lecture delivered in Queen's Hospital, Birmingham, (*Brit. Med. Jour.*,) exhibited a girl ten years old who, in being treated for subacute general chorea, had taken "Fowler's solution," in doses increasing from five to thirty-five minims, thrice daily. Not till then did toxic effects occur, and the chorea cease. After entire suspension of the drug for two days, it was continued for a time in doses of fifteen minims, and the chorea did not recur. The doctor said: "You may cautiously increase the dose of liquor arsenicalis far beyond the limits of the text-books, with the best results in chorea."

**Jequirity.**

The seeds which have been recently introduced under this name as a remedy in ophthalmic complaints, are derived from Abrus precatorius, a leguminous plant, indigenous to Africa and Southern Asia, and naturalized in tropical America. The hard seeds have a bright red integument, with a black spot surrounding the raphe. They are used in Oriental countries for ornaments and similar to beads; in Brazil they have been highly valued for several centuries in the treatment of certain diseases of the eye, an infusion being made of 32 powdered seeds (about 3 gm.) which are macerated for 24 hours with 500 gm. cold water, after which 500 gm. of hot water is added, and, when cooled, the liquid is filtered.

The results obtained by L. de Wecker show that this infusion produces conjunctivitis purulenta or cruposa as rapidly as inoculation, and that with due care, the desired inflammation may be well regulated. The experiments have not been concluded yet, and the active principle of the seeds is still unknown; an alkaloid prepared by Rigand & Dusart did not give similar good results, whether used by instillation or subcutaneously.—*Phar. Centralhalle.*

Another authority says: This remedy was first brought before European practitioners by DeWecker in the Annales d' Oculistique for July-August, 1882. The seeds of the jequirity plant, the Abrus precatorius, have been since a long time employed by the natives in some parts of Brazil in ocular affections. This method of treatment was first made known to De Wecker by one of his old patients, who, subsequently to his return home to Brazil from Paris, suffered a fresh outbreak of granular conjunctivitis, from which he was much relieved by the use of this drug.

The directions were to soak for twenty-four hours 32 grains of the powdered seeds in 1,000 grammes of water. The patient bathes his eyes with the filtered product thrice daily for three days, at the end of which time he has become the subject of a severe conjunctivitis, which may be either purulent or more allied to the diphtheritic form. By the fifteenth day the inflammation ceases, and the granulations are found to be much diminished in size, or even destroyed. [We have received a sample of the seeds above referred to, through the kindness of Prof. J. O. Stillson, ophthalmic surgeon to the Evansville (Ind.) Medical College; have applied it in one severe case of *traucoma* with excessive *pannus*, with most excellent results.—ED. BISTOURY]

---

**Hydrocele.**

In bringing this matter before the profession I feel bound to admit that, but for a curious accidental circumstance, the agent might never have presented itself to my notice. In the year 1875, I proposed to operate upon a patient aged 65, for the radical cure of a hydrocele of the tunica vaginalis. The disease had existed for about ten years, and had been repeatedly emptied by other surgeons. At this time I removed, by the trocar and cannula, about 12 ounces of serum, and, by accident, took from my pocket a bottle containing about two drachms of liquid ergotæ in the place of the same quantity of tincture of iodine, which it was my intention to throw into the cavity. On my return home, I discovered the mistake, and watched the patient for some hours at intervals. No inflammatory state occurred, and there was entire absence of pain, so that I allowed my patient to return to his ordinary occupation the next morning. To the present time there has been no return of the abnormal secretion. I have since, on two occasions, used the same plan with perfect success, and I attribute the cure to a specific action, exerted by ergot which re-establishes the balance between secretion and absorption.—J. E. W. Walker, M. R. C. S. E., in *British Medical Journal.*

---

**Distilled Water for Eye Lotions.**

In *The Practitioner*, Dr. Paul M. Chapman claims that it is not the best vehicle for eye lotions, saying: "I have tried the experiment on myself and on many of my friends, and the answer is always the same, viz., that the introduction of distilled water into the eye is attended with much discomfort and smarting, while with normal saline there is no noticeable effect whatever.

"The practical deduction is this, which I have also verified, that the addition of 2½ grains of chloride of sodium to the ounce of distilled water renders any lotion intended to be of a soothing character much more beneficial."

---

**Cholera Remedies.**

Here are the stock formulas published annually since the epidemic of 1866:

1. The "*Sun Remedy*," published in 1866 by the N. Y. *Daily Sun* and since then by almost every paper in the land. Every well-regulated clergyman in the country has it in his pocket-book for immediate reference: Two fluid drachms each of the tinctures of opium, camphor, capsicum, rhubarb and peppermint (concise and easily remembered). Take a teaspoonful in water every time the bowels move loosely.

(2) *Another.* Tinctures of opium, camphor, catechu, rhubarb, and peppermint, of each a fluid ounce; tincture of capsicum, half a fluid drachm.

(3) *Squibb's*, which is sometimes called *compound opium mixture*:

Chloroform, fl., ʒ 6.
Tincture of opium, fl. ℥ 2.
Spirit of camphor, fl. ℥ 2.
Tincture of capsicum, fl. ℥ 2.
Alcohol, sufficient to make fl. ℥ 10.

Dose 40 to 60 drops, taken in water each time the bowels move loosely.

It is important in any case of diarrhœa, but it is rarely appreciated except by physicians, that when there is too free action of the bowel, the patient should stop exercising and *lie down* flat on the back, and stay there as quietly as possible. If there is pain, simply apply heat; dry heat is best.

---

**White Agaric in the Sweating of Phthisis.**

Dr. Murell gives, in the *Practitioner*, an account of the action of white agaric (*Polyporus officinalis*) in the sweating of phthisis. After numerous experiments, he has arrived at the conclusion that it is a good remedy, and that there are times when it may be used with advantage: but he much doubts if it is equal to atropia, picrotoxin, pilocarpine, or Dover's powder. In small doses, it is uncertain in its action, whilst in larger doses it purges violently. The agaric was given in doses of three grains up to thirty. The drug in three-grain doses, acted slowly and not with certainty; three grains of extract, equal to nine grains of the powder, acting better, whilst thirty grains purged violently. A tincture of 1 in 6 and a fluid extract 1 in 3 have also been prepared. The active principle, agaricine has been given in the dose of $\frac{1}{12}$ of a grain, and was found to check cough and to promote sleep, as well as to restrain sweating.—*Pharmaceutical Journal.*

---

**Cellulose as a Dressing.**

Dr. Fischer, of Trieste, has made experiments with cellulose as a dressing to wounds, and has found it, when moistened with warm water or some medicated solution, and afterwards covered with an impervious fabric, to be a most excellent application in all cases where heat and moisture appear to be indicated. Its chief advantages are : 1. It is absolutely free from substances capable of exciting putrefaction. 2. It has a very low specific gravity. 3. It produces neither eczema nor erythema upon the epidermis. 4. It retains moisture and heat perfectly for more than twenty-four hours. 5. It never adheres to granulating wounds on the surface of the skin. 6. It adapts itself perfectly to the outline of the place of application. 7. It is much cheaper than other materials heretofore used for similar purposes.

Dr. Fischer has used, so far, only plain water or weak solution of carbolic acid or iodoform in the case of suppurating buboes, and has obtained uniformly satisfactory results.—*Zeitsch. f. Therap.*

---

**Local Anæsthetics.**

The following formulæ, from the *Medical News*, may be found serviceable as local anæsthetics for small operations:

Chloral hydrate.

Gum camphor, āā ʒij.

Chloroform, ʒj.

Morph. sul. ʒss.

Mix. This may be painted with a camel's hair brush over the area to be incised, allowed to dry and repeated as often as necessary to render the part insensible. Prof. Redier proposes the following:

Ether or chloroform, ʒ ij.

Camphor, ʒ ji.

Mix. Apply with a brush.

Crystallizable acetic acid, 1 part.
Chloroform, 20 parts.

Dissolve. Apply with a brush.

---

**Neuralgic Toothache.**

Dr. Von Kirchbauer claims to have had great success in treating toothaches that arise from neuralgia, with butyl chloral, or croton chloral. He takes:

Croton chloral, 1 drachm.
Glycerine, 6 drachms.
Distilled water, 2½ ounces.
Syrup of orange peel, 4 drachms.
Oil of fennel seed, 6 drops.

Dose, a tablespoonful, and if the pain continues very violent, repeat in an hour.

He applies carbolized and chloralized cotton locally. Equal parts of chloral hydrate and carbolic acid are put in a bottle, and when dissolved prepared cotton is saturated with it. The cotton may be left in the bottle until wanted for use, when a piece is cut off and rolled up in a pellet that is inserted in the cavity of the decayed tooth.

**Urinary Test Pellets, Dr. Piffard's Formula for.**

Sulphate of copper (ch. pure), 1 part.

Crystallized tartrate of sodium and potassium, 5 parts.

Sodic hydrate (ch. pure), 2 parts.

Mix thoroughly in a mortar; the more labor spent on this, the better the product. The result will be a pasty mass, which can be transferred to a wide-mouthed bottle and kept till wanted. To use it, take of the mass a piece about the size of a co. cath. pill, put it into a test-tube, and add about two fluid drachms of water; boil till the mass is dissolved, and the solution has a uniform pale and rather dirty-blue color; then add two or three drops of the suspected urine, and boil again for a moment. If sugar be present, the usual reaction will be apparent.

---

**Bacillus Malarial.**

In the *Clinique*, at Heidelberg, the following observations in regard to the bacillus of malaria were made:

Fresh blood obtained by the prick of a needle was employed to exhibit the living organisms, while uncolored preparations dried over a gas flame showed the bacilli deposited between the blood corpuscles. Such bacilli are about ¼ the size of a blood corpuscle, with both ends bulbous, and pale shaft. They possess power of locomotion. Four patients were examined in whom the bacilli were found. In one case, rendered doubtful owing to the presence of sugar in the urine, the bacilli disappeared entirely from the blood on the eleventh day of the use of quinine.—F. Ziehl, *Deut. Med. Wochenschrift.*

---

**Sweating of the Hands and Feet.**

Napthol has been recommended as an effective remedy against excessive sweating of the palms of the hands, foot-soles and axillas. These places should be moistened once or twice daily with a mixture of

Naphthol, 5 parts,
Glycerin, 10 parts.
Alcohol, 100 parts, and afterwards dusted, either with pure starch or with a mixture of

Naphthol, 2 parts.
Starch, 100 parts.

In the case of sweating feet, small pellets of antiseptic cotton are dipped in the powder and placed between the toes.—*Zeitsch. f. Diagn. and Ther.*

**Hiccough.**

Dr. C. S. Kellogg, in a recent number of the *Physician and Surgeon's Investigator*, directs attention to the value of the root of the pæonia officinalis, as an antispasmodic in the treatment of hiccough. In a case which he was called upon to treat he had tried in vain the remedies at hand, and was about to send for chloroform with which to control the spasm. Remembering that pæonia is claimed to be an antispasmodic he had the friends procure two ounces of the root, from which he prepared a decoction, which he gave with almost instant and permanent relief, the man taking only one-half of the quantity prepared. He has subsequently had occasion to try this remedy only once, but the result was equally prompt and satisfactory.—*Ther. Gaz.*

---

**Fissured Nipples.**

The man who will introduce a remedy which will either prevent, cure or render tolerable to the woman, fissured nipples, will win for himself a place in the affections of suffering womanhood. Many means of prophylaxis have been recommended, and the "sure cures" after the fissure has occurred, are so extremely numerous as to excite suspicion, it being a tolerably correct guide to the incurable nature of a disease when "sure cures" for its relief are abundant. *Le Practicien* contains another contribution to the sure cures of fissured nipples, by Monti. This consists of the solution of gutta percha in chloroform, just a sufficient quantity of the solvent being employed to make the solution fluid. The application having been made to the fissured nipple, the chloroform evaporates, leaving the gutta percha as a protecting pellicle, which does not come off through the sucking of the child.—*Med. Rec.*

---

**Diabetes.**

Diabetes has been a fruitful field for experiments. A great variety of remedies has been recommended for its relief, but it is as yet quite doubtful that any have been recommended which will meet the indications in all cases. The affection is apparently a very capricious one, yielding in some persons to remedies which were without effect in others. It therefore becomes the physician to have his repertory of agents for this particular affection well stocked. The latest addition thereto has been recommended in the form of iodoform, and is contained in a recent communication by Professor Moleschott to the Academy of Medicine, at Rome. He states that in cases of the disease in which he administered iodoform the quantity of sugar excreted rapidly diminished. Good results were observed to follow small doses in some cases, but usually the amount was increased to forty and fifty centigrammes daily before the characteristically beneficial results were observed. He used as a deodorizer of iodoform, cumarin (the odoriferous principle of the tonko bean.)—*Ther. Gaz.*

